# Integrated curation and data mining for disease and phenotype models at the Rat Genome Database

**DOI:** 10.1093/database/baz014

**Published:** 2019-02-11

**Authors:** Shur-Jen Wang, Stanley J F Laulederkind, Yiqing Zhao, G Thomas Hayman, Jennifer R Smith, Monika Tutaj, Jyothi Thota, Marek A Tutaj, Matthew J Hoffman, Elizabeth R Bolton, Jeffrey De Pons, Melinda R Dwinell, Mary Shimoyama

**Affiliations:** 1 Department of Biomedical Engineering, Marquette University and Medical College of Wisconsin, Milwaukee, WI, USA; 2 Department of Physiology, Medical College of Wisconsin, Milwaukee, WI, USA

## Abstract

Rats have been used as research models in biomedical research for over 150 years. These disease models arise from naturally occurring mutations, selective breeding and, more recently, genome manipulation. Through the innovation of genome-editing technologies, genome-modified rats provide precision models of disease by disrupting or complementing targeted genes. To facilitate the use of these data produced from rat disease models, the Rat Genome Database (RGD) organizes rat strains and annotates these strains with disease and qualitative phenotype terms as well as quantitative phenotype measurements. From the curated quantitative data, the expected phenotype profile ranges were established through a meta-analysis pipeline using inbred rat strains in control conditions. The disease and qualitative phenotype annotations are propagated to their associated genes and alleles if applicable. Currently, RGD has curated nearly 1300 rat strains with disease/phenotype annotations and about 11% of them have known allele associations. All of the annotations (disease and phenotype) are integrated and displayed on the strain, gene and allele report pages. Finding disease and phenotype models at RGD can be done by searching for terms in the ontology browser, browsing the disease or phenotype ontology branches or entering keywords in the general search. Use cases are provided to show different targeted searches of rat strains at RGD.

## Introduction

The laboratory rat (*Rattus norvegicus*) has been used as an animal model in biomedical research for over 150 years. The first recorded inbreeding of a rat strain for scientific purposes was started by King in the early 1900s (1). Since then, there have been increasing numbers of rat models from naturally occurring mutations, selective breeding and, more recently, genome manipulation, including chemical mutagenesis and genome editing. Through the innovation of genome-editing technologies (2) and rat embryonic stem cells (3), genome-modified rats provide precision models of disease by disrupting or complementing targeted genes. The genome-edited models not only confirm the causal effect of targeted genes but also can be used as *in vivo* models to further study disease pathogenesis and treatment. These genome-modified rats, and selectively bred rats and their parental strains, have been used in a wide range of research in the fields of physiology, pharmacology, toxicology, nutrition, behavior, immunology and pathology. As a result, there are more than 1.6 million publications of rat research in PubMed, with about 35 000 being added every year. The vast amount of data embedded in these publications has great value to scientists in future research planning. The Rat Genome Database (RGD; http://rgd.mcw.edu) was created in 1999 to organize the existing knowledge and present it to the research community with integrated genomic, genetic, phenotypic and disease datasets.

To facilitate integration of and access to rat data, RGD uses multiple ontologies to standardize data and present these datasets in a format that can be read by humans and machines. RGD’s primary focus is manual annotation of genes, strains and quantitative trait loci (QTL). Particularly noteworthy are the manually curated disease annotations across rat, human and mouse genes at RGD. The primary annotations are made by curators from peer-reviewed journals and transferred to the other two genes of the ortholog group (4). Automated pipelines are set up to bring in data from other databases to complement the RGD manually curated disease-gene data. RGD regularly imports disease data from the ClinVar database (5), OMIM (6) and the Comparative Toxicogenomics Database (7), as well as maintaining archival disease annotations from the Genetic Association Database (8).

To make rat strain data easy to find, RGD established the Rat Strain (RS) Ontology to depict relationships among strains (9). The RS Ontology organizes rat strains into different strain types based on their breeding history and genetic backgrounds. Using the rat strain as a data hub, users can navigate among different datasets associated with the strain of interest. These datasets include manually curated disease annotations, qualitative mammalian phenotype (MP) annotations and quantitative phenotype annotations.

## Results

### Strain registration

**Figure 1:**
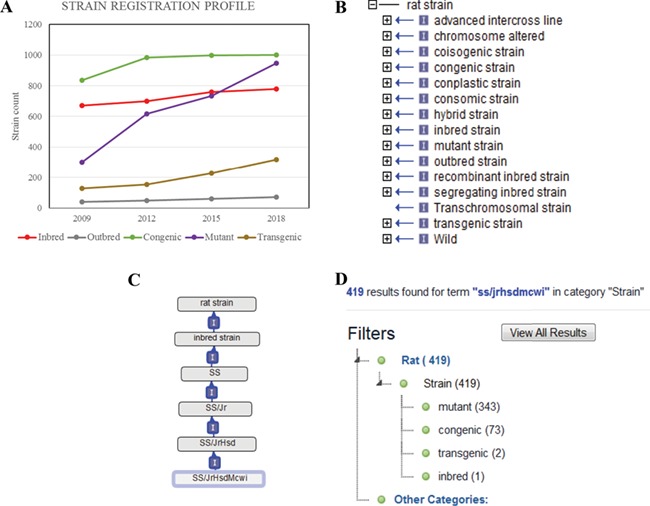
(A) The total counts of the registered rat strains at RGD over a 10-year period. Five strain types, inbred, outbred, congenic, mutant and transgenic strains, are selected to show the growth trends among different strain types. (B) The 15 high-level nodes of the RS Ontology. (C) The ontological tree of SS/JrHsdMcwi. (D) Using SS/JrHsdMcwi as a search keyword to retrieve strains related to this strain. The numbers in parentheses show the total number and the breakdown numbers for the strain types.

RGD currently has a catalogue of more than 3000 registered strains and substrains. These strains were curated from publications and submissions by authors and vendors. RGD regularly receives new strain submissions from major rat resources such as the Gene Editing Rat Resource Center (https://rgd.mcw.edu/wg/gerrc/), the Rat Resource and Research Center (http://www.rrrc.us/), the National BioResource Project (http://www.anim.med.kyoto-u.ac.jp/nbr/Default.aspx) in Japan and commercial rat providers. Researchers can submit their strains and obtain the official symbols and IDs used for manuscript publication. The Strain Submission Form (https://rgd.mcw.edu/rgdweb/models/strainSubmissionForm.html?new=true) can be found via the ‘Submit Data’ link on the RGD homepage (http://rgd.mcw.edu/) or on the menu bar of the Strain Search page, which can be accessed from the link in the center of the RGD homepage. Since the launch of the RS in 2009, there has been a steady increase in registered strains (Figure 1A). Of all strains, close to one-third are congenics, an inbred host carrying a particular locus from a donor strain. The congenics were used extensively to identify chromosomal elements associated with a particular phenotype in early genomic studies. The number of mutant strains, which include rats carrying spontaneous mutations or induced mutations, has almost tripled between 2009 and 2018. The transgenic strains, rats carrying foreign DNA sequence, are another strain type that shows steady increase since 2009.

### RS Ontology

**Figure 2:**
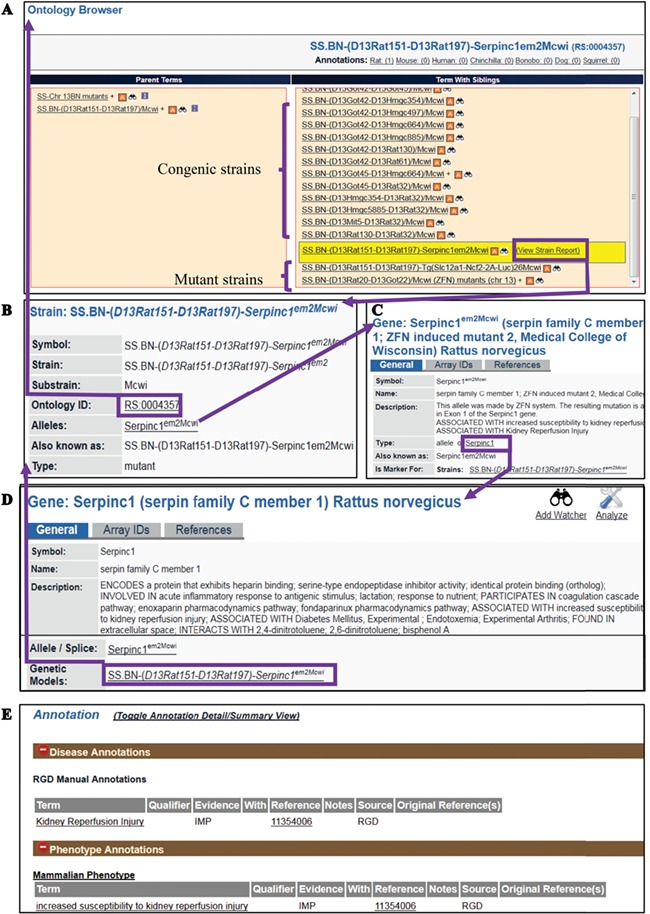
(A) The RS Ontology browser displaying the selected mutant strain ‘SS.BN-(D13Rat151-D13Rat197)-*Serpinc1^em2Mcwi^*’ with its congenic and mutant siblings listed in the same column and its parents on the left. The browser provides the link to the rat strain report page (B), which displays ontology ID and associated data. The report pages for strain (SS.BN-(*D13Rat151-D13Rat197)-Serpinc1^em2Mcwi^*), allele (Serpinc1^em2Mcwi^) (C) and the parent gene (Serpinc1) (D) are integrated by hyperlinks to each other. Annotations (E) are found in all the report pages and are expanded to show links to the curated terms and original references.

The RS Ontology includes 15 first-level nodes depicting the strain types (Figure 1B). Each strain type is named following standardized nomenclature rules (9). The breeding history and genome modification information are embedded in the tree structure. For example, the salt-sensitive Sprague–Dawley (SS) rat strain was developed by L.K. Dahl (10) and then distributed to different institutions where three substrains were developed: SS/Jr, SS/Hsd and SS/N. SS/JrHsdMcwi was originally derived as SS/Jr, then bred at Harlan (Hsd), and is currently housed at the Medical College of Wisconsin (Figure 1C). Searching RGD with SS/JrHsdMcwi as a keyword returns 419 related strains, including mutants, congenics, transgenics and inbreds (Figure 1D). Using the Ontology Browser, users can find rat strains by how they were generated or from which parents they were derived. Considering SS.BN-(*D13Rat151-D13Rat197)-Serpinc1^em2Mcwi^* (RGD: 12790721, RS:0004357) as an example (Figure 2A). Its two ontological parents SS.BN-(D13Rat151-D13Rat197)/Mcwi (RS:0001711) and SS-Chr 13BN mutants (RS:0004542) to the left of it in the browser depict that the strain is derived from SS.BN-(D13Rat151-D13Rat197)/Mcwi and is a mutant strain derived from SS congenics introgressed with Chr13 DNA fragment from Brown Norway. Strains with sibling relationship are co-listed in the same column, which includes congenic and mutant strains. The Strain Report page containing all RGD data related to the strain is accessed via the ‘View Strain Report’ link on the browser page. The Strain Report page (Figure 2B) contains links to go back to the Browser, to the mutant allele Serpinc1^em2Mcwi^ (Figure 2C) carried by the strain, and other basic information about the strain. The mutant allele created by gene-editing techniques is named according to standard nomenclature rules and is linked to its parent gene report page and its associated rat strain report page. Each mutant allele and the allele-carrying strain are annotated with diseases and/or phenotypes, and the annotations are propagated to the parent gene Serpinc1. These manual annotations are integrated in the database and can be found in the related pages linked by the gene. For example, the strain SS.BN-(*D13Rat151-D13Rat197)-Serpinc1^em2Mcwi^* has been found to be more susceptible to kidney reperfusion injury than the control wild-type, based on the study by Wang *et al.* (11). The curated diseases and phenotypes were integrated into the Serpinc1^em2Mcwi^ mutant allele page, the Serpinc1 gene page and the mutant rat strain page. Users can find these annotations in the Annotation section (Figure 2E) of all three report pages, and each page provides links to the others.

**Figure 3:**
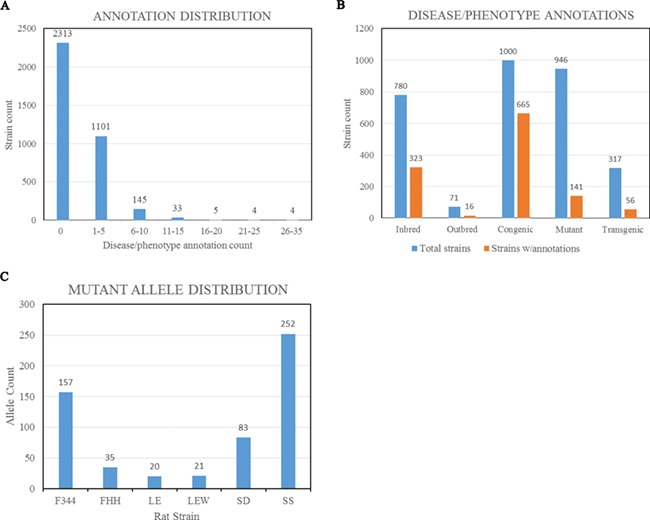
(A) Disease/phenotype distribution among rat strains at RGD. There are total 3605 registered strains shown in the bar chart. Close to 35% of the strains are annotated with one or more disease/phenotype terms. (B) Bar chart comparing the numbers of rat strains with disease/phenotype annotations across the selected five strain categories. (C) Rat strains carrying mutant alleles are grouped by background strains. Strain groups carrying at least 20 alleles are presented. F344, Fischer 344; FHH, Fawn Hooded Hypertensive; LE, Long Evans; LEW, Lewis; SD, Sprague–Dawley; SS, salt-sensitive Sprague–Dawley.

**Figure 4:**
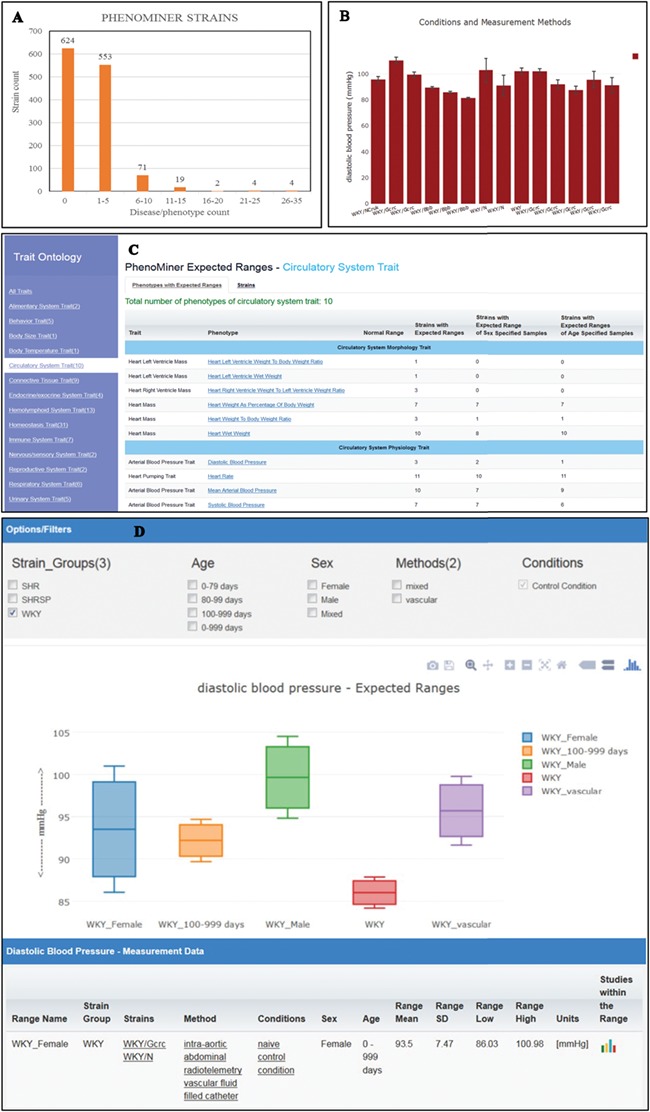
(A) The disease/phenotype annotation distribution of strains with PhenoMiner data. More than half of the PhenoMiner strains have at least one disease/phenotype qualitative annotation. (B) The result page in PhenoMiner after querying WKY strains for diastolic blood pressure, using specific measurement methods under control conditions. The bar chart displays all the matched records in the PhenoMiner database. (C) Summary of the traits for expected ranges analysis is listed on the left and the list of the available expected ranges under the selected circulatory system trait is shown in blue font. Each phenotype is hyperlinked to the associated expected ranges data. (D) The expected ranges of diastolic blood pressure of the selected WKY strain group. Each color-coded box is an interquartile range graph in which the data median is shown as the line in the middle, the third quartile is shown as the top and first quartile is shown as the bottom of the box. Maximum value is represented by the horizontal bar above the box and the minimum value is represented by the horizontal bar below the box. The calculated expected ranges data associated with the displayed graphs are listed at the bottom of the graph, and the links to the original PhenoMiner records are also provided.

### Functional annotations: disease and phenotype

The Rat has been a preferred model for complex disease research such as cardiovascular, metabolic syndrome and neurobehavioral studies. To understand the underlying molecular mechanisms of disease, researchers have been inbreeding rats, generating congenic/consomic strains and engineering rat genes to create strains with different disease manifestations or susceptibilities. These strains, either susceptible or resistant to the targeted diseases/phenotypes, are annotated with standardized RGD Disease Ontology (RDO) (4) (12) or MP Ontology (13) terms. These functional annotations are listed in the Annotation section on all the report pages for strains, genes and alleles and can be expanded to view details such as evidence codes and citations (Figure 2E). In the expanded view, each annotation is listed with the original reference, evidence code, optional qualifier and free text notes. About 35% of the strains (~1300 strains) are annotated with one or more disease/phenotype terms (Figure 3A). Among strain types, over 65% of congenic strains are annotated, while less than 20% of transgenics and mutants are annotated (Figure 3B). We anticipate more publications will be generated from the recently created gene-modified strains in the near future. These gene-modified strains carrying defined alterations in target genes are ideal tools for disease study. By strain group, the SS group carries the most mutant alleles, followed by F344 (Figure 3C). There are some genes that have multiple alleles carried by different host rat strains. The rat Lepr gene (RGD:3001) has the highest number of mutant alleles found in different genetic backgrounds. These mutant alleles include the spontaneous mutant alleles Lepr^fa^, Lepr^cp^ and Lepr^m1Rll^ and engineered mutant alleles Lepr^em1^, Lepr^em2^, Lepr^em3^ and Lepr^em2Mcwi^. These allele-carrying strains are annotated with diseases and/or phenotypes, and the annotations are propagated to the parent gene Lepr.

**Figure 5:**
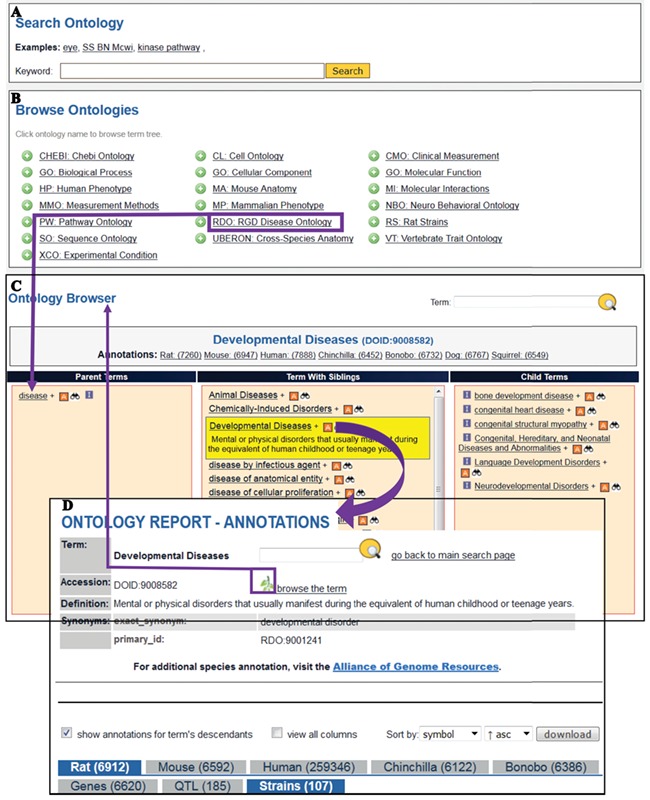
The Search Ontology (A) and Browse Ontologies (B) functions allow users to retrieve all strains annotated to a particular term. (C) The selected term ‘Developmental Diseases’ and its definition are highlighted in yellow in the center column with its child terms listed in the right column. The ‘A’ icon is a link to the ontology report page (D) listing all RGD objects annotated to the disease term ‘Developmental Diseases’ and its child terms. The links provided in the browser page and the ontology report page allow users to navigate between the terms and annotations. Strains associated with developmental diseases can be downloaded from the download button above the species tabs.

### PhenoMiner Expected Range

In addition to qualitative disease and phenotype curation, RGD also curates rat strains with quantitative phenotype annotations through the PhenoMiner tool (https://rgd.mcw.edu/rgdweb/phenominer/home.jsp) (14), which are accessed through the PhenoMiner user interface tool (15). The PhenoMiner annotations, like disease/phenotype annotations, are accessible from the Annotation section on the strain report page. Currently, there are more than 1200 strains curated with PhenoMiner annotations and more than half of these PhenoMiner strains are also annotated with disease or phenotype terms (Figure 4A). The PhenoMiner user interface query is built from combinations of the four ontologies (16) used for curation. The tool retrieves all the matched quantitative phenotype records in the database and displays them as a bar chart with the specified clinical measurement (Figure 4B). The result gives researchers an overview of the value range curated at PhenoMiner and can be used as a reference in study planning. To provide more utility of PhenoMiner data, RGD launched a new project, PhenoMiner Expected Ranges, to perform statistical meta-analysis on PhenoMiner data. The aim of this project is to provide an expected range of a phenotype measurement based on the records in the database. For example, the diastolic blood pressure records displayed in Figure 4B show all the diastolic blood pressure records from the WKY strain group. Statistical tests were applied to these records to decide how they could be grouped to calculate expected ranges with statistical significance. Several exploratory analyses were conducted on publications and numbers of experiments in the database to determine inclusion/exclusion of individual studies/observations in the analysis. The heterogeneity in the meta-analysis was examined by Cochrane’s Q and I^2^, which were then used as the threshold to choose the appropriate meta-analysis model for each dataset. The statistics theories and computational algorithm are described in the accompanying paper by Zhao *et al*. in this issue. The PhenoMiner Expected Ranges tool (https://rgd.mcw.edu/rgdweb/phenominer/phenominerExpectedRanges/views/home.html) can be accessed from the Phenotypes and Models icon on the RGD homepage. In the tool, the meta-data are grouped by the Vertebrate Trait Ontology (17) listed on the left panel (Figure 4C). Once a trait is selected, the table of available expected ranges of the phenotypes within the selected trait is displayed. The most comprehensive datasets are under the circulatory system trait, where 10 phenotypes are available for 11 inbred strain groups and 200 expected ranges were calculated. Diastolic blood pressure, one of the phenotypes under the trait, has expected ranges calculated from three strains groups, SHR, SHRSP and WKY, and only calculated ranges from WKY were selected to display (Figure 4D). Each color-coded box represents an interquartile range in which the data median is shown as the line in the middle, the third quartile is presented by the top and the first quartile by the bottom. If enough records are available in the database, usually more than four records per phenotype, the expected ranges will also be calculated with different stratifications such as age, sex or methods of measurement. This graph displays the expected ranges for overall WKY group, WKY older than 100 day-old or just male or female specific ranges. The result displayed can be modified by applying filters such as age, sex and methods. At present, only measurements made under control conditions were used in the analysis. The data table following the graph display provides users with the details on the strains, methods, conditions, sex and age groups associated with the displayed graph. The links to original PhenoMiner data are also provided in the far right column of the data table.

### Finding disease/phenotype rat models

To find data objects annotated with ontology terms, users can either search the terms by keywords or browse the selected ontology. The searching and browsing of ontologies (Figure 5 A&B) is accessible from the Ontologies icon on the RGD homepage. The RDO is currently used for disease curation (4) (12), and MP (13) is used for rat-strain phenotype curation.

#### Finding disease models by browsing the Disease Ontology

The RGD ontology browser launched in 2012 (18) uses three columns to display all the parent terms on the left and all the child terms on the right of the selected term. When the disease browser is opened for browsing the Disease Ontology, the top level term ‘disease’ is shown in the browser with child terms listed on its right. The term Developmental Diseases (DOID: 9008582) is a direct child of ‘disease’ and selection of this term moves it to the center column with the parent term ‘disease’ on the left and all the child terms on the right as shown in Figure 5C. Terms having child terms are displayed with a ‘+’ icon, and the ‘A’ icon to the right of developmental diseases means that annotations for the term itself and/or its child term(s) exist in RGD. The ‘A’ icon is a link to the Ontology report-annotations page listing all RGD objects annotated to that term. There are more than 6900 rat genes, 185 rat QTL and 107 rat strains annotated with ‘Developmental Disease’ or any of its more specific child terms (Figure 5D). The strain-associated disease annotations are viewed under the ‘Strains’ tab. Users can view all the annotation-associated data such as strain symbols, chromosome positions of the genes, evidence codes used in making the annotations, references and notes by clicking the ‘view all columns’ box. Annotations can be downloaded in the ‘view all columns’ table format and analysed in a spreadsheet. The non-redundant lists of developmental disease rat models presented in Table 1 were selected annotations manually curated from publications. The superscript portion of strain symbols reveals whether the strain is a spontaneous mutant (*m*) such as WAG-*F8^m1Ycb^* (RGD: 2314904) carrying a spontaneous mutation of the rat F8 gene or a genome-modified mutant such as SD/Novo-*F8^em1Sage−/−^* (RGD: 11531091) carrying an endonuclease-mediated (*em*) mutation of F8. The strain report page has a link to the allele report page where the details of the allele and the link to its parent gene are available.

#### Finding disease models by searching the MP Ontology

Using ‘abnormal lymphocyte morphology’ as the search term in the ontology search (Figure 5A) retrieved Human Phenotype terms, MP terms and their child terms. The MP term ‘abnormal lymphocyte morphology (MP:0002619)’ was selected to retrieve rat strains that were annotated with this ontology term and its child terms at RGD. Annotations can be viewed by going to the Ontology Report-Annotations page (MP:0002619) via the ‘A’ icon next to the MP term in the search results. Strains are downloaded and processed by similar mechanisms used for the developmental disease strains shown in Table 1. The non-redundant list of phenotype models is presented in Table 2. All the strains in Table 2 carry identified mutant alleles that are associated with the abnormal phenotypes. Among them, Rag1 mutant alleles cause abnormal phenotypes in B cells, T cells and NK cells.

**Table 1:** Rat strains with associated developmental disease terms are listed with curated disease terms, official strain symbols and the known disease genes/alleles with representative references. The downloaded strain data are processed by selecting non-redundant disease annotations with manual evidence codes. The associated alleles not included in the downloaded file can be found on the strain report pages

Disease term	Disease ID	Official strain symbol	Rat strain RGD ID	Disease-associated gene		Reference	
				Parent gene	Mutant allele	PMID	RGD ID
Albinism	DOID:9001386	DA-*Tyr ^em1Kyo^*	8552298	Tyr	Tyr^em1Kyo^	23409244	12792973
Amelogenesis imperfecta	DOID:2187	WT/Jtt	2303759	Unknown	Unknown	17710440	2303758
Ataxia telangiectasia	DOID:12704	F344-*Atm ^em1Kyo^*	12879400	Atm	Atm^em1Kyo^	28007901	12879399
Attention deficit disorder with hyperactivity	DOID:1094	Wig/Ymas	4891165	Unknown	Unknown	17610585	4891167
Autism spectrum disorder	DOID:0060041	SD-*Nlgn3 ^em1Sage^*	11568700	Nlgn3	Nlgn3^em1Sag^e	24773431	9831152
		SD-*Fmr1 ^em1Sage^*	11568040	Fmr1	Fmr1^em1Sage^	24773431	9831152
Canavan disease	DOID:3613	TRM/Kyo	1302702	Aspa	Deletion	10820213	628404
Hypertrophic cardiomyopathy	DOID:11984	SS-Chr 16^BN^/Mcwi	629524	Unknown	Unknown	17204904	1598977
Charcot–Marie–Tooth disease	DOID:10595	SD-Tg(Pmp22)Kan	2312447	Mouse Pmp22	n/a	8630243	2312445
Cryptorchidism	DOID:11383	SS-*Adamts16 ^em1Bj^*	13437612	Adamts16	Adamts16^em1Bj^	24983376	13434925
		KH	2301330	Unknown	Unknown	6140035	2301322
		LE/OrlBarth	10047391	Unknown	Unknown	26502805	12911229
		SDLEF7/Barth	10047393	Unknown	Unknown	26502805	12911229
Dwarfism	DOID:9007661	SDR/Slc	1302698	Gh1	Gh1^sdr^	2752987	1578505
		WIC-*Tg ^rdw^* /Kts	2304039	Tg	Tg^rdw^	11089535	730133
Gray platelet syndrome	DOID:0111044	WF/NHsd	737908	Unknown	Unknown	2040691	11531117
		SD/Novo-*F8 ^em1Sage−/−^*	11531091	F8	F8^em1Sage^	24931420	11530071
Hemophilia A	DOID:12134	WAG-*F8 ^m1Ycb^*	2314904	F8	F8^m1Ycb^	20626616	7245964
Hermansky–Pudlak syndrome	DOID:3753	TM/Kyo	1302623	Rab38	Rab38^ru^	15112108	1300411
		FHH	60993	Rab38	Rab38^ru^	15112108	1300411
Learning disorders	DOID:8927	SS.SR-*(D17Rat24-rs106534785)* /Opaz	7401201	Unknown	Unknown	23469157	7394833
Megacolon and its children	DOID:11372	AR-Ednrbsl/Hkv	6480218	Ednrb	Ednrb^sl^	22132166	6480215
		LE/Hkv.AR-*Ednrb ^sl^*	6480220	Ednrb	Ednrb^sl^	21915282	6480217
		F344.AR-*Ednrbsl* /Hkv	6480223	Ednrb	Ednrb^sl^	22132166	6480215
		Sl	629492	Ednrb	Ednrb^sl^	8589685	1342447
Microcephaly	DOID:10907	WI-*Cit ^fhJjlo^* /Rrrc	6482243	Cit	Cit^fhJjlo^	10219263	13204836
Microphthalmia	DOID:10629	SHR-*Gja8*^m1Cub^	2293729	Gja8	Gja8^m1Cub^	18470322	2293186
		SD-*Pax6 ^Sey^* /Mce	2325754	Pax6	Pax6^Sey^	7981749	1601213
		UPL		Unknown	Unknown	8282038	727242
Muscular dystrophy, Duchenne	DOID:11723	SD-*Dmd ^em1Ang^*	12880037	Dmd	Dmd^em1Ang^	25310701	12880034
		W-*Dmd ^em1Kykn^*	10045593	Dmd	Dmd^em1Kykn^	25005781	11040981
Polydactyly	DOID:1148	SHR.PD-(*D8Rat42-D8Arb23*)/Cub	1641851	Zbtb16	Zbtb16^Lx^	19191224	2312786
Polycystic kidney diseases and its children	DOID:9002730	SPRD-*Anks6 ^PKD^* /Rrrc	1302377	Anks6	Anks6^PKD^	7933831	1300446
		PKD	68118	Anks6	Anks6^PKD^	9097967	629573
		PKD/Mhm	11535000	Anks6	Anks6^PKD^	16207829	11534987
		SD-Tg(hCMV-Anks6^PKD)^Mhm	11535031	Anks6	Anks6^PKD^	21119215	7207426
		PCK-*Pkhd1 ^pck^* /CrljCrl	1580542	Pkhd1	Pkhd1^pck^	11919560	70439
Retinitis pigmentosa	DOID:10584	SD-Tg(Rho^*^P23H)1Lav	1358298	Mouse Rho	P23H mutant	26009893	11065783

**Table 2:** Rat strains with the associated MPs are listed with phenotype terms, official strain symbols and the known associated genes/alleles with representative references. The downloaded strain data are processed by selecting non-redundant phenotype annotation with manual evidence codes. The associated alleles not included in the downloaded file can be found on the strain report pages

MP	Phenotype ID	Rat strain symbol	Rat strain RGD ID	Phenotype-associated gene		Reference	
				Parent gene	Mutant allele	PMID	RGD ID
Decreased B cell number	MP:0005017	LEW-*Rag1 ^em1Ztm^*	7204133	Rag1	Rag1^em1Ztm^	23136839	7204131
		SS-*Rag1 ^em1Mcwi^*	4139884	Rag1	Rag1^em1Mcwi^	23364523	7207429
		SD-*Rag1 ^em1Ang^*	7204136	Rag1	Rag1^em1Ang^	23150522	7204134
		SS-*Cd247 ^em1Mcwi^*	6484582	Cd247	Cd247^em1Mcwi^	24343121	13442481
Decreased NK cell number	MP:0008045	SD-*Il15 ^em1Soar^*	11087551	Il15	Il15^em1Soa^	28395334	12910490
Decreased T cell number	MP:0005018	F344-*Atm ^m1Kyo^*	12879394	Atm	Atm^m1Kyo^	27895165	12879393
		LEW-*Rag1 ^em1Ztm^*	7204133	Rag1	Rag1^em1Ztm^	23136839	7204131
		SD-*Lep ^em1Sage^*	12904912	Lep	Lep^em1Sage^	22948215	12904911
		SS-*Cd247 ^em1Mcwi^*	6484582	Cd247	Cd247^em1Mcwi^	24343121	13442481
		SS-*Rag1 ^em1Mcwi^*	4139884	Rag1	Rag1^em1Mcwi^	23364523	7207429
		SD-*Rag1 ^em1Ang^*	7204136	Rag1	Rag1^em1Ang^	23150522	7204134
Increased double-positive T cell number	MP:0005091	F344-*Atm ^m1Kyo^*	12879394	Atm	Atm^m1Kyo^	27895165	12879393
Increased lymphocyte cell number	MP:0005013	SS-*Sh2b3 ^em2Mcwi−/−^*	5686766	Sh2b3	Sh2b3^em2Mcwi^	25628389	12904914
Increased regulatory T cell number	MP:0004973	SS-*Sh2b3 ^em1Mcwi−/−^*	5686318	Sh2b3	Sh2b3^em1Mcwi^	25776069	13442483

#### Finding disease models by gene family in the general search

**Figure 6:**
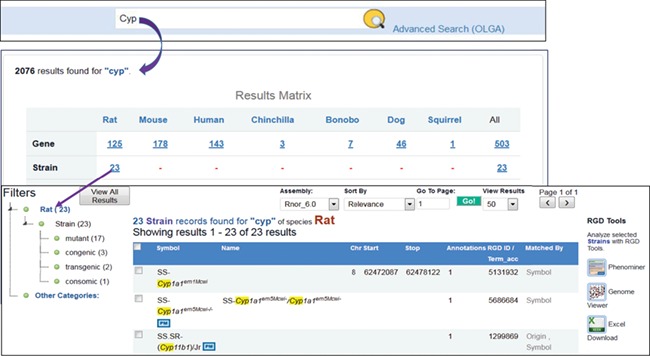
Finding rat strains associated with the cytochrome P450 superfamily. Using the keyword ‘cyp’ in the general search retrieves RGD data associated with the gene family. Returned results are displayed in a matrix with species as the column titles. There are 23 rat strains associated with the gene family.

RGD’s general search tool has been redesigned in 2018 with open source technologies Java and Elasticsearch, a real-time distributed full-text search and analytics engine built on top of Apache Lucene. The search box is placed at the top center of every webpage. To find strains associated with the cytochrome P450 superfamily, users enter ‘cyp’ as the search term in the box and the search engine will retrieve all the entries matched ‘cyp’ in the description, object symbols, origins, synonyms and other indexed terms. The results (Figure 6) are aggregated into groups and presented in a matrix across species, data objects, ontology terms and references. There are 23 strains matched with ‘cyp’ by name, symbol, synonyms or origins. These 23 strains include mutant, congenic, transgenic and consomic strains, and their tallies are shown next to strain types on the following page accessed by clicking the ‘23’. The data can be downloaded as an Excel file or sent to other RGD tools such as Phenominer or Variant Visualizer for further analysis. Each strain is hyperlinked to its report page where details of the strain and its associated annotations can be found. The strains tagged with ‘PM’ icons have PhenoMiner annotations available at RGD.

### Data accessibility

RGD is actively participating in the NIH Data Commons Initiative. RGD’s Application programming interface (APIs) were developed according to the SmartAPI specifications and have been registered at smart-api.info with the tag ‘NIHdatacommons’ to conform to the Data Commons FAIR standard. To be specific, RGD’s strain-related data are available programmatically via a few REST API endpoints (https://rest.rgd.mcw.edu/rgdws/swagger-ui.html). Users can obtain data for a single strain by RGD ID, or for all strain records in RGD. ‘Mapped strains’, such as congenic strains where the genomic position of the introgressed region has been curated, can be retrieved by querying by chromosome, start and stop position and a Map Key that designates the appropriate genome assembly. Some of the API endpoints provide functionality to retrieve strains annotated to specific disease, phenotype or RS Ontology terms or to retrieve the annotations for a specific strain. RGD’s PhenoMiner quantitative phenotype data are also accessible via a REST API endpoint. By submitting a combination of accession IDs for Clinical Measurement, Experimental Condition, Rat Strain and/or Measurement Method ontology terms, users can access the measurement values and associated metadata that match their query.

## Conclusion

Using strain as a data object, RGD curators annotate experimental rat models with disease and phenotype terms. Curated strains can be searched by genes, diseases or phenotypes, and the results can be imported into RGD tools or downloaded for customized analysis. To facilitate data navigation, annotations are integrated among genes, strains and associated mutant alleles. Users can identify the disease strain, disease-causing allele and its parent gene beginning on one of the report pages. For more than a decade, RGD has undertaken a focused curation effort aimed at capturing comprehensive disease and phenotype data of studied rat strains. In addition to using qualitative terms to curate phenotypes, RGD has moved phenotype curation to the quantitative level by developing the PhenoMiner tool. From these curated quantitative phenotype data, Zhao *et al.* (accompanying paper in this issue) have developed the Expected Range tool to allow researchers to visualize the phenotype profiles across multiple strains under similar experimental conditions from multiple sources. The combination of expected range strain profiles and the disease/phenotype annotations of each individual strain can inform researchers whether the strain is a good disease model for the targeted disease and how much deviation of the phenotype the strain exhibits as compared to the normal range of rat strains.

Rat disease models have evolved from spontaneous and randomly induced mutants to targeted mutants generated by advanced genome-editing techniques. The targeted mutants are powerful tools to dissect pathogenesis at the molecular level. However, the disease manifestation of a mutant allele typically also depends on other host genes. Rats in which the same mutation has been engineered in different genetic backgrounds are valuable tools to study how the genomic context plays a role in disease manifestation. Since the publication of the reference genome for the Brown Norway rat in 2004 (19), many other rat strains have also been sequenced (20). RGD houses genomic variation data, relative to Rnor3.4, Rnor5.0 and Rnor6.0 reference genomes, for more than 40 rat strains and these data are available for various browsing and analysing tools (21). The combination of annotations with genome variation profiles among rat strains provides robust utility for translational genomics.

## Notes

**Database URL**: https://rgd.mcw.edu/
